# EPC‐derived exosomes promote osteoclastogenesis through LncRNA‐MALAT1

**DOI:** 10.1111/jcmm.14228

**Published:** 2019-04-25

**Authors:** Yigong Cui, Shenglong Fu, Dong Sun, Junchao Xing, Tianyong Hou, Xuehui Wu

**Affiliations:** ^1^ Department of Orthopaedics Southwest Hospital The Third Military Medical University Chongqing P.R. China; ^2^ Department of Orthopaedics Jinan Fifth People's Hospital Shandong P.R. China

**Keywords:** bone marrow‐derived macrophages, bone repair, endothelial progenitor cells, ITGB1, LncRNA‐MALAT1, miR‐124, osteoclastogenesis

## Abstract

Bone repair involves bone resorption through osteoclastogenesis and the stimulation of neovascularization and osteogenesis by endothelial progenitor cells (EPCs). However, the role of EPCs in osteoclastogenesis is unclear. In this study, we assess the effects of EPC‐derived exosomes on the migration and osteoclastic differentiation of primary mouse bone marrow‐derived macrophages (BMMs) in vitro using immunofluorescence, western blotting, RT‐PCR and Transwell assays. We also evaluated the effects of EPC‐derived exosomes on the homing and osteoclastic differentiation of transplanted BMMs in a mouse bone fracture model in vivo. We found that EPCs cultured with BMMs secreted exosomes into the medium and, compared with EPCs, exosomes had a higher expression level of LncRNA‐MALAT1. We confirmed that LncRNA‐MALAT1 directly binds to miR‐124 to negatively control miR‐124 activity. Moreover, overexpression of miR‐124 could reverse the migration and osteoclastic differentiation of BMMs induced by EPC‐derived exosomes. A dual‐luciferase reporter assay indicated that the integrin ITGB1 is the target of miR‐124. Mice treated with EPC‐derived exosome‐BMM co‐transplantations exhibited increased neovascularization at the fracture site and enhanced fracture healing compared with those treated with BMMs alone. Overall, our results suggest that EPC‐derived exosomes can promote bone repair by enhancing recruitment and differentiation of osteoclast precursors through LncRNA‐MALAT1.

## 1 INTRODUCTION

The process of bone healing occurs appropriately in the majority of cases through the formation of callus.^1^ However, some fractures fail to heal which can result in delayed union or persistent non‐union and prolonged disability.^2^ There are various causes of failed bone repair including inadequate blood supply, inappropriate angiogenesis, insufficient immobilization and infection.^3^, ^4^, ^5^ In particular, the initiation of bone repair involves appropriate bone resorption through osteoclastogenesis.^6^, ^7^ Osteoclasts originate from the precursors of macrophage/monocyte lineage and are cells that specialize in bone resorption.^8^ The osteoclast is responsible for removing the organic and inorganic components of bone and is critical for normal bone function.^9^ Osteoclastogenesis is induced by the expression of numerous genes, including *MMP9*,* CTSK*,* TRAP* and *CAR2*.^10^

Endothelial progenitor cells (EPCs) are believed to promote bone repair by stimulating neovascularization and osteogenesis.^10^, ^11^, ^12^ In the absence of appropriate vascularization, hypoxia and the disruption of biomechanical signalling pathways can prevent the regeneration of bone tissue.^6^ Moreover, the process of bone formation is thought to be coupled to the process of angiogenesis.^7^, ^8^ Pro‐angiogenic factors secreted by bone cells, such as VEGF, can trigger signalling responses from various cells expressing VEGF receptors, which include endothelial cells and osteoclasts.^9^ EPCs contribute to the formation of new blood vessels and indirectly contribute to the formation of new bone during bone repair.^13^ It has been proposed that endothelial cell‐specific and cell‐autonomous Notch activity regulates bone angiogenesis and couples it to osteogenesis, possibly by interactions with VEGF.^14^

Non‐coding RNAs are generally allocated into two major classes based on size, with those over 200 nucleotides referred to as long non‐coding RNAs (lncRNAs) and the others called microRNAs (miRNAs).^15^ Non‐coding RNA possess no apparent protein‐coding capability but participate in various biological and pathological processes and a number have been implicated in osteogenesis.^16^, ^17^, ^18^ In previous research, microRNA‐124 (miR‐124) was found to regulate osteoclast differentiation of mouse bone marrow‐derived macrophages (BMMs) by suppressing nuclear factor of activated T cell, cytoplasmic 1 (NFATc1) expression.^19^ miR‐124 was also found to negatively regulate osteogenic differentiation and bone formation in mesenchymal stem cells by interacting with Dlx transcription factors, which play an important role in osteoblast differentiation.^20^ Several studies have implicated that lncRNAs dysregulate miRNAs through competitive binding.^21^, ^22^ Lnc‐MALAT1 is thought to competitively regulate miR‐124 to promote EMT and the development of non‐small cell lung cancer.^23^ Therefore, lncRNA MALAT‐1 may influence the role of miR‐124 in osteogenesis. Integrin subunit β 1 (ITGB1) is thought to be the target of miR‐124. Interestingly, ITGB1 was found to be the major and essential β integrin receptor for insulin‐like growth factor‐binding protein 1 (IGFBP1) and is thought to be responsible for its pro‐osteoclastogenic function.^24^

In a preliminary study, we found that EPCs enhance the migration and osteoclastic differentiation of BMMs in vitro and in a mouse femur fracture model. However, the role of EPCs in osteoclast formation and function is still unclear. EPC‐derived exosomes have been found to participate in the communication between EPCs and bone marrow stromal cells to promote osteoblastic differentiation by inhibiting the expression of osteogenic genes and increasing proliferation in vitro.^25^ In this study, we examined the effects of EPC‐derived exosomes on the migration and osteoclastic differentiation of primary mouse BMMs in vitro. We also evaluated the effects of EPC‐derived exosomes on the homing and osteoclastic differentiation of transplanted BMMs in a mouse bone fracture model in vivo.

In summary, the aim of this study was to test the hypothesis that EPC‐derived exosomes may promote osteoclastogenesis through the Lnc‐MALAT‐1/miR‐124 pathway.

## 2 MATERIALS AND METHODS

### 2.1 Animals

Sixty C57BL/10 mice (6 weeks old) were obtained from the Model Animal Research Center (MARC) of Nanjing University. All animal experiments were conducted in accordance with the Institutional Guidelines for the Care and Use of Laboratory Animals of the Southwest Hospital, affiliated with the Third Military Medical University. Study protocols were reviewed and approved by the Animal Ethics Committee of the Southwest Hospital. Animals were randomly divided into OVX and Control groups, n = 30 in each.

### 2.2 Isolation of EPCs and BMMs

To isolate EPCs, cells expressing the early EPC surface marker CD133 were selected from mouse umbilical cord blood using anti‐CD133‐coupled magnetic microbeads (Miltenyi Biotech, Bergisch Gladbach, Germany). The EPCs were then cultured in DMEM supplemented with 10% foetal bovine serum (FBS). Monoclonal antibodies against CD31, CD34, CD133, vWF and UFA‐1 (Sigma‐Aldrich, St. Louis, MO) were used to conduct immunophenotypic analysis on the EPCs with Isotype‐identical antibodies as controls (PharMingen, San Diego, CA). The carbocyanine fluorescent dye Dil (Molecular Probes, Eugene, OR) was used to counterstain cells. The angiogenic capacity of early EPCs was determined by a Matrigel tube formation assay as described in our previous report.^26^ In brief, Matrigel (Sigma‐Aldrich) was diluted in 500 μl EGM‐2 media (1:1 v/v) in 96‐well plates and incubated at 37°C for 1 hour to allow polymerization. EPCs (2 × 10^4^ cells/well) were seeded onto the Matrigel and incubated at 37°C for 24 hours. Digital micrographs were taken for morphological analysis.

### 2.3 BMMs isolated from mouse calvariae

Primary BMMs were isolated from the whole bone marrow. Briefly, 6‐week‐old C57BL/10 mice were killed by rapid decapitation under deep anaesthesia with 10% chloral hydrate, marrow was extracted from femora and tibiae and placed in Petri dishes in a culture medium composed of α‐minimal essential medium (α‐MEM) supplemented with 10% inactivated FBS, 100 IU/mL penicillin G and 100 μg/mL streptomycin and CMG medium (conditioned medium containing 100 ng/mL mouse M‐CSF) in a 1:10 ratio. Cells were incubated at 37°C with 5% CO_2_ for 3 days. When the medium was changed, the cells were washed to deplete residual stromal cells. After reaching 90% confluence, the cells were washed with PBS three times and trypsinized for 30 min to harvest BMMs. Non‐adherent cells were layered onto a Ficoll density gradient solution and centrifuged at 440 *×* g for 30 minutes at room temperature. Cells lying in the upper layer were harvested as BMMs. To identify osteoclasts, cells were fixed in 4% paraformaldehyde for 20 minutes and stained for tartrate‐resistant acid phosphatase (TRAP) using a commercial kit (Sigma‐Aldrich). TRAP‐positive multinucleated cells containing three or more nuclei were considered mature osteoclasts and counted.

### 2.4 Isolation and identification of EPC‐exosomes

Medium collected from EPCs was centrifuged to remove cellular debris (2500 × g for 15 minutes at 4°C) and filtered with a 0.22‐μm filter (Merck‐Millipore, Burlington, MA). The filtered solution was transferred to a 15 mL Amicon Ultra‐15 Centrifugal Filter Unit (Merck‐Millipore) and centrifuged at 4000 × g until the volume in the upper compartment containing the exosomes was reduced to 200 μL. The exosomes were washed three times by suspending in PBS followed by centrifugation. The washed filtrate containing the exosomes was laid on top of a 30% sucrose/D_2_O cushion in a sterile Ultra‐Clear™ tube (Beckman Coulter, Brea, CA) and ultracentrifuged at 100 000 × g for 1 hour at 4°C. The pellets were resuspended in 15 mL PBS and centrifuged at 4000 × g until the volume was concentrated to approximately 200 μL. The total number of exosomes was determined using a CD63 ExoELISA™ kit (System Biosciences, Palo Alto, CA) following manufacturers’ instructions. Exosomes were identified by dynamic light scattering analysis and transmission electron microscopy (TEM). RNA and proteins were extracted for further analysis from exosomes using a Total Exosome RNA & Protein Isolation Kit (Invitrogen, Carlsbad, CA).

### 2.5 Transwell migration assay

Bone marrow‐derived macrophages (1 × 10^5^/well) were loaded into the upper chamber in MEM‐α media with 5% FBS and EPCs into the lower chamber of Transwell inserts with a pore size of 8 μm (Costar, NY). After incubation (37°C with 5% CO_2_ for 5 days), the BMMs were rinsed with PBS, fixed in 10% formalin for 10 minutes and stained with DAPI for 15 minutes. The migration capacity of the BMMs was determined under an inverted light microscope as described previously.^27^ Cells that had migrated to the lower surface of the membrane were counted in three randomly selected fields.

### 2.6 Cell transfection

For Lnc‐MALAT1 expression analysis, siRNA against the Lnc‐MALAT1 vector was synthesized by GenePharma (Shanghai, China). EPCs were transfected with Lnc‐MALAT1 down‐regulation vector at a final concentration of 50 nM using Lipofectamine 2000 (Invitrogen, Carlsbad, CA) according to the manufacturer's protocol.

For miR‐124 overexpression, an miR‐124 mimic or corresponding negative control (miR‐NC) was obtained from GenePharma (Shanghai, China). BMM cells were transfected with either miR‐124 mimic or miR‐NC at a final concentration of 50 nM using Lipofectamine 2000 (Invitrogen) according to the manufacturer's protocol. Cells were used for miR‐124 expression analysis or other experiments after 48 h of transfection.

For ITGB1 analysis, an ITGB1 vector was constructed by GenePharma (Shanghai, China) then BMM cells were transfected with either ITGB1 vector or NC at a final concentration of 50 nmol/L using Lipofectamine 2000 (Invitrogen) according to the manufacturer's protocol.

### 2.7 Luciferase reporter assay

HEK293T cells were cotransfected with plasmids containing 3′‐UTR of wild or mutant fragments from ITGB1 or predicted binding sequence from Lnc‐MALAT1 and miRNA mimics using Lipofectamine 2000 (Invitrogen) according to the manufacturer's protocol. Forty‐eight hours after transfection, firefly and Renilla luciferase activities were measured consecutively by using a dual‐luciferase reporter assay system (Promega, Fitchburg, WI). Ratios of luminescence from firefly to Renilla luciferase were calculated and each assay was repeated in three independent experiments.

### 2.8 Quantitative real‐time PCR analysis

RNA was isolated from BMMs or bone tissues using TRIzol (Invitrogen). cDNA was synthesized from 1 μg of total RNA in 21‐μl reaction volumes using oligo dT18 primers and SuperScript reverse transcriptase. PCR amplification was carried out with Taq DNA polymerase (TaKaRa, Tokyo, Japan) using 1 μl of the first‐strand cDNAs as templates. The amplification reactions were run with 30 thermocycles of 30 seconds at 94°C, 30 seconds at 55°C and 30 seconds at 72°C.

### 2.9 Ribonucleoprotein immunoprecipitation (RIP) assay

Anti‐AGO2 ribonucleoprotein immunoprecipitation (RIP) was performed in HEK‐293T cells transfected with miR‐124 mimics or miR‐NC. Briefly, HEK‐293T cell lysates were pre‐blocked with Protein G beads (Invitrogen) and then incubated with anti‐AGO G beads (Pierce Biotechnology, Waltham, MA) at 4°C for 90 minutes. Beads were collected by centrifugation at 600 × g for 1 minute, washed 5 times with RIPA buffer and resuspended in Tris‐HCl 50 mmol/L (pH 7.0). The beads were then incubated 45 minutes at 70°C to reverse the crosslinks and the RNAs that co‐IP with anti‐AGO antibodies were extracted using TRIzol (Invitrogen) following the manufacturer's instructions and then quantified by RT‐PCR.

### 2.10 RNA interference

Bone marrow‐derived macrophages were collected and resuspended in Electroporation Isoosmolar Buffer (Eppendorf). The cells were transfected using electroporation with a Stealth RNAi™ small interfering RNA (siRNA) targeting Lnc‐MALAT1 (sense sequence: CAGCUCAUUGCUGGCUACAUAGAUA, Invitrogen) or a Stealth RNAi™ siRNA negative control (NC) on an ECM830 Electro‐Square Porator (Harvard Apparatus). The electroporation was carried out using a single square wave pulse of 2500 V/cm field strength with 300 μs pulse length. Cells were then allowed to recover for 46 hours in DMEM.

### 2.11 Western blot analysis

Proteins (50 μg) from lysed cells were separated by 10% SDS‐PAGE and transferred to nitrocellulose membranes. After blocking for 2 hours, the membranes were incubated overnight with primary antibodies followed by horseradish peroxidase (HRP)‐conjugated secondary antibodies. The protein bands were visualized using enhanced chemiluminescence. Densitometry analysis of protein levels was performed with Gel‐pro Image Analysis Software (Media Cybernetics, Rockville, MD, USA).

### 2.12 Femur fracture model

The fracture model involved 80 male mice (12‐13 weeks old, mean body weight 250‐300 g). Mice were anaesthetized with intraperitoneal ketamine hydrochloride (60 mg/kg) and xylazine hydrochloride (10 mg/kg) before surgical procedures took place under aseptic conditions. Femur fractures (unilateral) were produced by 3‐point bending as described by Manigrasso and O'Connor^28^ with unfractured femurs serving as controls. Of the 80 mice used in the study, eight mice were excluded due to poor fracture quality. Immediately following the fracture, mice received intravenous injections of Dil‐labelled BMMs (2 × 10^5^ cells) suspended in Endothelial Basal Medium‐2 (EBM‐2, Lonza, Basel, Switzerland), either alone or in combination with EPC‐derived exosomes (236 471.5 pg/mL) (n = 20 for each). To detect their ability to home into the fracture site, BMMs were labelled with Dil (2.5 mg/mL; Molecular Probes) for 5 minutes at 37°C followed by 15 minutes at 4°C prior to transplantation. To assess angiogenesis by EPC‐derived exosomes, tissue samples were collected from the fracture site on day 7 or day 28 after cell transplantation. Samples were embedded in OCT compound (Sakura Finetek Japan, Tokyo, Japan) and cut into 5‐μm thick sections. The sections were stained with anti‐CD31 antibody (Vector Laboratories, Burlingame, CA), counterstained with DAPI and examined under a fluorescence microscope. Dil‐positive capillaries were counted in 10 randomly selected high‐power fields.

### 2.13 Microcomputed tomography

The trabecular volumes at the distal femoral metaphysis and proximal tibia were determined using a Scanco CT40 scanner (Scanco Medical AG, Bassersdorf, Switzerland). Approximately, 100 slices were analysed, beginning at the point where the condyles and primary spongiosa were no longer visible.

### 2.14 Statistical analysis

All data are reported as the mean ± SD. The Student's two‐tailed unpaired *t* test was used to determine differences between two groups. A *P *< 0.05 was regarded as statistically significant.

## 3 RESULTS

### 3.1 EPCs promote the migration and osteoclastic differentiation of BMMs through exosomes

**Figure 1:**
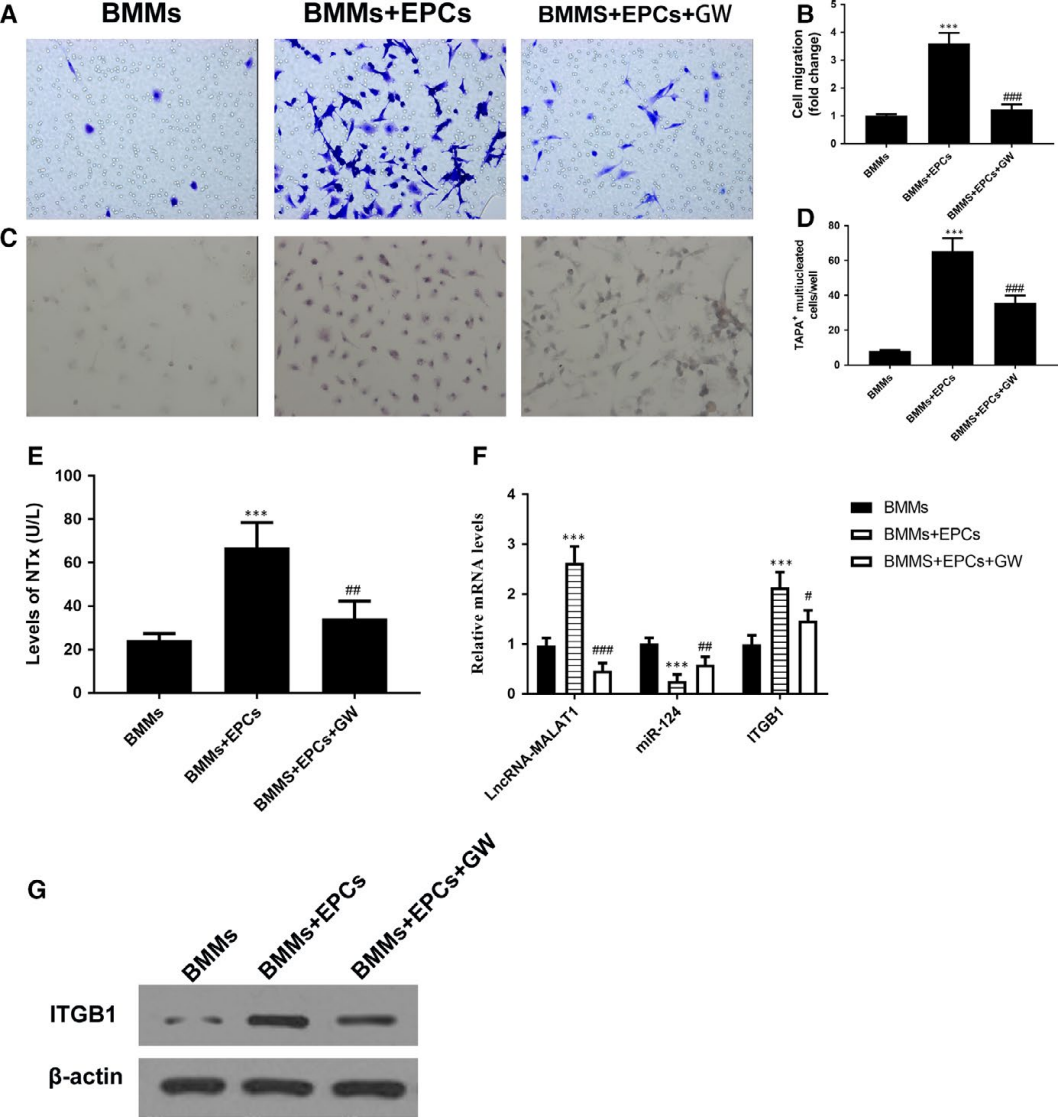
Endothelial progenitor cells (EPCs) promote the migration and osteoclastic differentiation of co‐cultured bone marrow‐derived macrophages (BMMs) through exosomes. (A and B) Representative images of BMMs (stained with crystal violet) on the lower surface of a Transwell membrane (A) and quantitative analysis of cell migration by cell counting (B) after 5 d in culture. Cells are either cultured alone (control) or co‐cultured with EPCs (co‐culture) or co‐cultured with EPCs and with exosomal inhibitor GW4869. (C and D) Representative immunofluorescence images of tartrate‐resistant acid phosphatase (TRAP)‐stained cells (C, magnification 200×) and the numbers of TRAP‐positive multinucleated (≥3 nuclei) cells (D) of BMMs after 7 d of culture. The cells were either cultured alone (control), co‐cultured with EPCs (co‐culture) or co‐cultured with EPCs and added exosomal inhibitor GW4869 (Co+GW). E, The levels of NTx tested by ELISA (F) The mRNA levels of lncRNA‐MALAT1, miR‐124 and ITGB1 by quantitative RT‐PCR and the protein levels of ITGB1 by western blot analysis (G) in BMMs after 7 d of culture. n = 3, ***P < 0.001 vs control, ^#^ *P* < 0.05, ^##^ *P* < 0.01 ^###^ *P* < 0.001 vs co‐culture

The influence of EPCs on the migration and osteoclastic differentiation of BMMs was analysed by a Transwell assay and quantitative analysis of cell migration by cell counting. In BMMs cultured alone or in medium conditioned with EPCs, there was a distinct difference in the appearance of the cells after 5 days in culture (Figure 1A,B). Migration and osteoclastic differentiation significantly increased in the cells cultured in the medium conditioned with EPCs (*P *< 0.001 vs control) but when an exosomal inhibitor (GW4869) was added to the media the level of migration and differentiation decreased (*P* < 0.001 vs co‐culture) (Figure 1C,D). Differentiation was measured by the number of TRAP‐positive multinucleated (≥3 nuclei) BMMs. We also measured for bone resorption using biomarkers, such as N‐telopeptides (NTx), by ELISA using Novocalcin and Pyrilinks‐D kits from Metra Biosystems (Minneapolis, MN). The result showed that the level of NTx in BMMs cultured in the medium conditioned with EPCs was significantly higher compared to BMMs cultured alone (Figure 1E). The mRNA levels of lncRNA‐MALAT1, miR‐124 and ITGB1 were then evaluated in BMMs grown alone or in the medium conditioned with EPCs (Figure 1F). Quantitative RT‐PCR indicated a significantly higher level of lncRNA‐MALAT1 and ITGB1 in BMMs cultured in the medium conditioned with EPCs (*P* < 0.001 vs control), which corresponded with a significantly lower level of miR‐124 (*P* < 0.001 vs control). The addition of GW4869 led to a reduction in the levels of lncRNA‐MALAT1 and ITGB1 (*P* < 0.001 vs co‐culture), whereas levels of miR‐124 were increased (*P* < 0.001 vs co‐culture). Similar results were observed in protein levels of ITGB1 by western blot analysis (Figure 1G).

**Figure 2:**
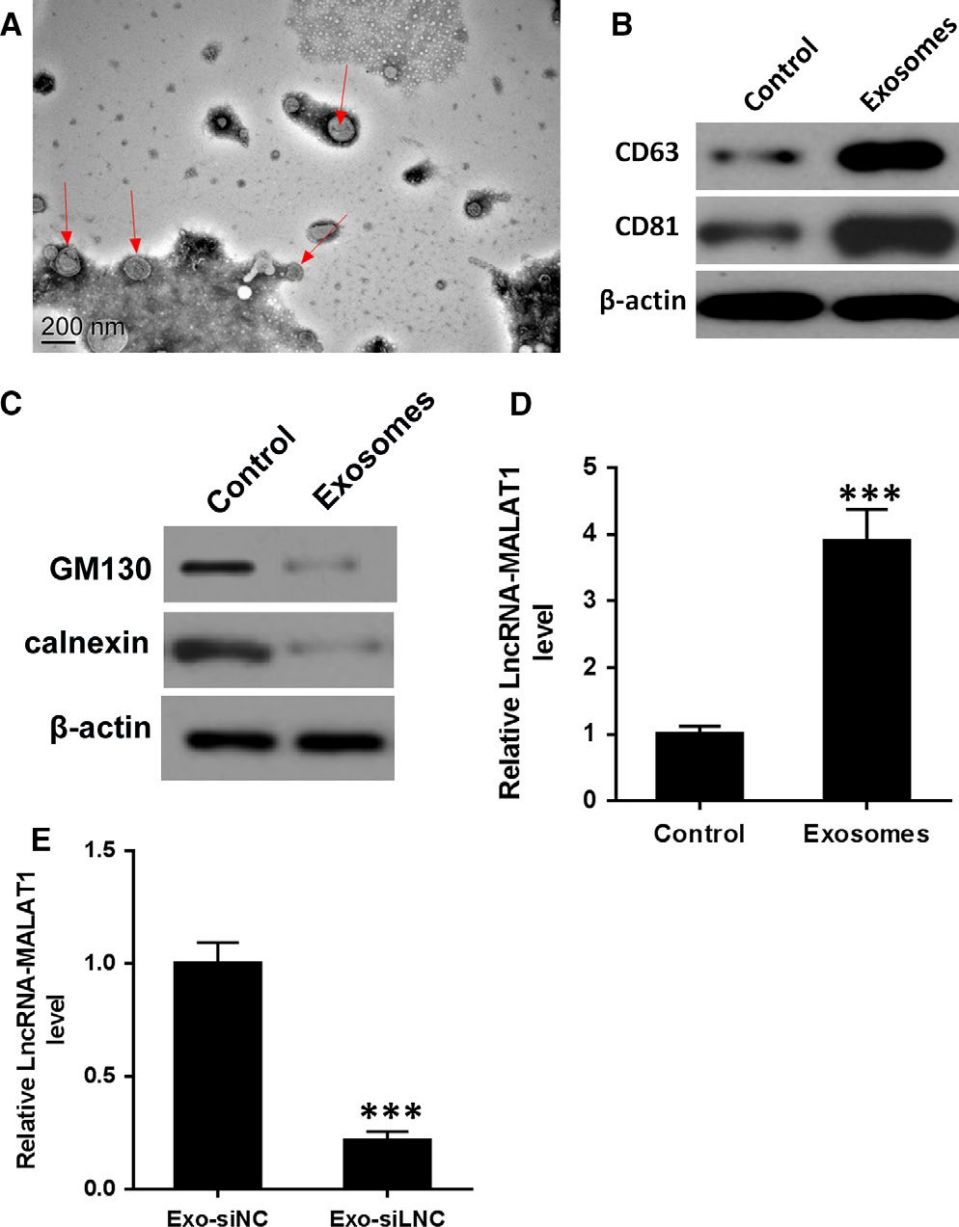
Identification of endothelial progenitor cells (EPC)‐derived exosomes. A, Exosomes extracted from EPCs were identified by transmission electron microscopy (TEM). Magnification: ×150 000. Scale bar: 200 nm. (B‐C) the protein levels of CD63. CD81, GM130 and calnexin by western blot analysis in EPC‐derived exosomes. D, The mRNA levels of lncRNA‐MALAT1 in EPCs and EPC‐derived exosomes. E, The mRNA levels of lncRNA‐MALAT1 in EPC‐derived exosomes transfected with a lncRNA‐MALAT1‐targeting siRNA prior or negative control. ***P < 0.001 vs control

The concentration of exosomes in the solution was about 1.35 × 10^9^/mL and identified in EPCs by TEM (Figure 2A). Western blotting using the positive exosomal markers CD63 and CD81 (Figure 2B) and negative exosome markers GM130 and calnexin (Figure 2C) were also used to identify exosomes. Levels of lncRNA‐MALAT1 mRNA were found to be significantly higher in EPC‐derived exosomes than in EPCs (Figure 2D) and lncRNA‐MALAT1 expression was inhibited in EPC‐derived exosomes transfected with a lncRNA‐MALAT1‐targeting siRNA (Figure 2E).

Overall, these results indicate that exosomes released from EPCs give rise to increased levels of migration and osteoclastic differentiation in BMMs. Moreover, EPC‐derived exosomes increased the levels of lncRNA‐MALAT1 and ITGB1 in BMMs but decrease the levels of miR‐124.

### 3.2 MALAT‐1 competitively regulated miR‐124

**Figure 3:**
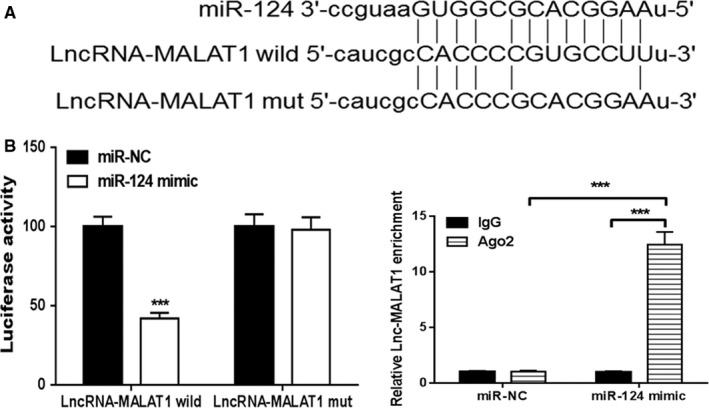
MALAT‐1 can regulate miR‐124. A, Schematic representation of the predicted binding sites for miR‐124 and the site mutagenesis design for the reporter assay. B, A luciferase reporter plasmid containing wild‐type or mutant lncRNA‐MALAT1 was cotransfected into HEK‐293T cells with miR‐124 or miR‐NC. Luciferase activity was determined at 48 h after transfection using the dual‐luciferase assay and was normalized to Renilla activity. C, Anti‐AGO2 ribonucleoprotein immunoprecipitation (RIP) was performed in HEK‐293T cells transfected with miR‐124 mimics or miR‐NC, followed by RT‐PCR to detect lncRNA‐MALAT1. ***P < 0.001 vs miR‐NC

A putative binding site for miR‐124 was mutated in lncRNA‐MALAT1 and the binding of lncRNA‐MALAT1 to miR‐124 was confirmed in HEK‐293T cells by assessing luciferase activity (Figure 3A,B). Anti‐AGO2 ribonucleoprotein immunoprecipitation was performed in HEK‐293T cells transfected with miR‐NC or miR‐124 mimic, followed by an assessment of lncRNA‐MALAT1 levels by RT‐PCR. Increased levels of lncRNA‐MALAT1 in the RIP of cells transfected with miR‐124 mimic confirmed the interaction between miR‐124 and lncRNA‐MALAT1 (Figure 3C).

### 3.3 Exosomal LncRNA‐MALAT1 induces osteoclastic differentiation in vitro

**Figure 4:**
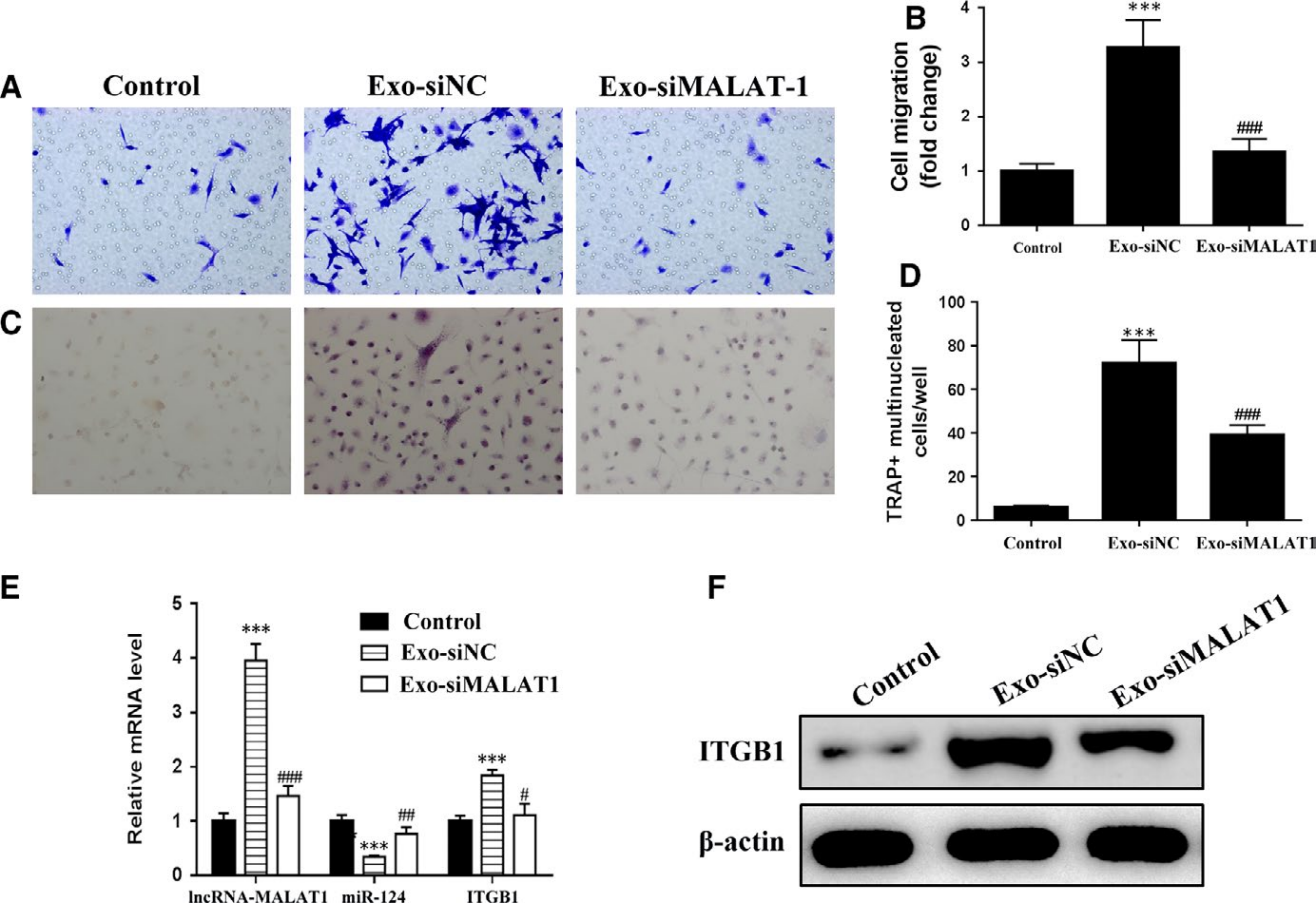
Exosomes of lncRNA‐MALAT1 could induce osteoclastic differentiation. (A and B) Representative images of BMMs (stained with crystal violet) on the lower surface of the Transwell membrane (A) and quantitative analysis of cell migration by cell counting (B) after 5 d in culture, with the cells either cultured alone (control) or treated with endothelial progenitor cells (EPCs) transfected with negative control‐derived exosomes (Exo‐siNC) or treated with EPCs transfected with lncRNA‐MALAT1‐targeting siRNA‐derived exosomes (Exo‐siMALAT1) for 24 h. (C and D) Representative immunofluorescence images of tartrate‐resistant acid phosphatase (TRAP) stained cells (C, magnification 200×) and the numbers of TRAP‐positive multinucleated (≥3 nuclei) cells (E) of bone marrow‐derived macrophages (BMMs) after 7 d of culture, with the cells either cultured alone (control) or treated with EPC transfected with negative control‐derived exosomes (Exo‐siNC) or with EPCs transfected with lncRNA‐MALAT1‐targeting siRNA‐derived exosomes (Exo‐siMARAT1) for 24 h. F, The mRNA levels of lncRNA‐MALAT1, miR‐124 and ITGB1 by quantitative RT‐PCR and the protein levels of ITGB1 by western blot analysis (G) in BMMs after 7 d of culture. n = 3, ***P < 0.001 vs control, ^#^ *P* < 0.05, ^##^ *P* < 0.01, ^###^ *P* < 0.001 vs Exo‐siNC

Migration was assessed in BMMs treated for 24 hours with either EPC‐derived exosomes transfected with NC (Exo‐siNC) or lncRNA‐MALAT1‐targeting siRNA (Exo‐siMALAT1). Migration was significantly reduced in control BMMs and cells transfected with Exo‐siMALAT1 (*P* < 0.001 vs Exo‐siNC); however, migration was increased in cells treated with NC‐derived exosomes (*P* < 0.001 vs control) (Figure 4A,B). TRAP staining in the same BMMs treated for 24 hours with either Exo‐siNC or Exo‐siMALAT1 revealed that osteoclastic differentiation was less induced by Exo‐siMALAT1 than by siNC (*P* < 0.001 vs Exo‐siNC) (Figure 4C,D). Osteoclastic differentiation was almost absent in untreated BMMs. The relative mRNA levels of lncRNA‐MALAT1, miR‐124 and ITGB1 were evaluated in BMMs either grown alone or treated with Exo‐siNC or Exo‐siMALAT1. The expressions of lncRNA‐MALAT1 and ITGB1 were elevated in Exo‐siNC‐treated BMMs, however, the expression of miR‐124 was reduced (*P* < 0.001 vs control). Silencing lncRNA‐MALAT1 attenuated this effect. The expressions of lncRNA‐MALAT1 and ITGB1 in Exo‐siMALAT1‐treated BMMs were reduced compared to Exo‐siNC‐treated BMMs, however the expression of miR‐124 was elevated (Figure 4E). There were also higher levels of ITGB1 protein in BMMs treated with Exo‐siNC compared to Exo‐siMALAT1 with almost undetectable levels of ITGB1 in untreated BMMs (Figure 4F). These results indicate that an interaction exists between miR‐124 and lncRNA‐MALAT1, which has consequences on the migration and osteoclastic differentiation of BMMs.

### 3.4 Exosomes induced osteoclastic differentiation through inhibition of miR‐124

**Figure 5:**
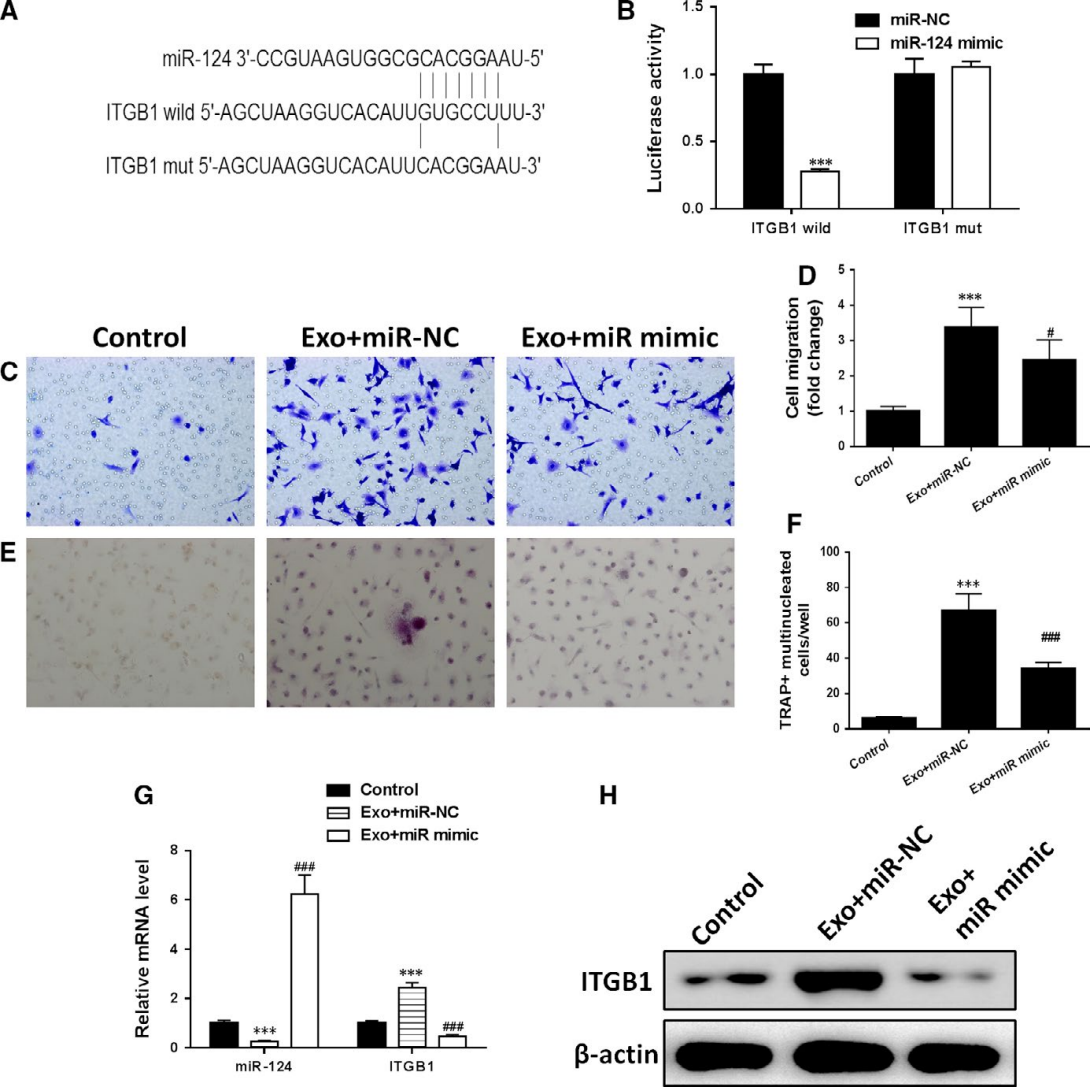
Exosomes induced osteoclastic differentiation through inhibition of miR‐124. A, miR‐124 potential binding sites on the 3′‐UTR of ITGB1. B, A luciferase reporter plasmid containing wild‐type or mutant ITGB1 was cotransfected into HEK‐293T cells with miR‐124 or miR‐NC. Luciferase activity was determined at 48 h after transfection using the dual‐luciferase assay and was normalized to Renilla activity. (C and D) Representative images of bone marrow‐derived macrophages (BMMs), stained with crystal violet, on the lower surface of the Transwell membrane (C) and quantitative analysis of cell migration by cell counting (D) after 5 d in culture. The BMMs were either cultured alone (control) or transfected with miR‐124 mimic and treated with EPC‐derived exosomes or transfected with negative control and treated with EPC‐derived exosomes for 24 h. (E and F) Representative immunofluorescence images of tartrate‐resistant acid phosphatase (TRAP)‐stained cells (C, magnification 200×) and the numbers of TRAP‐positive multinucleated (≥3 nuclei) cells (F) of BMMs after 7 d of culture, with the BMMs either cultured alone (control) or transfected with miR‐124 mimic and treated with EPC‐derived exosomes or transfected with negative control and treated with EPC‐derived exosomes for 24 h. G, The mRNA levels of miR‐124 and ITGB1 by quantitative RT‐PCR and the protein levels of ITGB1 by western blot analysis (H) in BMMs after 7 d of culture. n = 3, ***P < 0.001 vs control, ^#^ *P* < 0.05, ^###^ *P* < 0.001 vs Exo+mimic NC

We next assessed whether a potential miR‐124 binding site on the 3′‐UTR of ITGB may influence migration of osteoclastic differentiation. The potential 3′‐UTR binding site was mutated and an interaction between miR‐124 and ITGB1 was confirmed through a luciferase reporter assay in HEK‐293T cells (Figure 5A,B). The previous migration and osteoclastic experiments conducted with lncRNA‐MALAT1‐targeting siRNA (Figure 4A‐D) were repeated with a similar set of experiments for miR‐124 mimic (Figure 5C‐F). BMMs were either transfected with miR‐124 mimic or an NC and treated for 24 hours with EPC‐derived exosomes. The migration of the BMMs treated with exosomes increased when they were transfected with NCs (*P* < 0.001 vs control) (Figure 5C). However, BMMs transfected with miR‐124 mimics and treated with EPC‐derived exosomes migrated less than the NC but more than the untreated control (*P* < 0.05 vs exosomes + miR‐124 mimic). TRAP staining was performed after 7 days of culture to determine the status of osteoclastic differentiation in the treated BMMs. Osteoclastic differentiation was increased in BMMs transfected with the NC and treated with EPC‐derived exosomes (*P* < 0.001 vs control) (Figure 5F). Transfection with miR‐124 mimic reduced osteoclastic differentiation in BMMs but levels remained higher than in BMMs untreated with EPC‐derived exosomes (*P* < 0.001 vs exosomes + NC). The mRNA levels of miR‐124 and ITGB1 were measured by quantitative RT‐PCR (Figure 5G). When the levels of miR‐124 mRNA increase the levels of ITGB1 are reduced. The protein levels of ITGB1 by western blot analysis in BMMs substantiate these findings (Figure 5H). Higher levels of miR‐124 result in lower levels of ITGB1.

**Figure 6:**
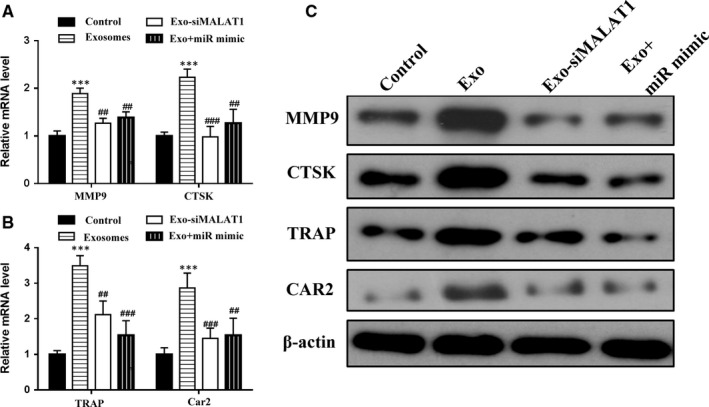
Endothelial progenitor cells (EPCs) promote osteoclast gene expression in bone marrow‐derived macrophages (BMMs) through exosomes. (A and B) The mRNA levels of *MMP9*,*CTSK*,*TRAP*, and *CAR2* by quantitative RT‐PCR and the protein levels of these genes by western blot analysis (C) in BMMs after 7 d of culture. The BMMs were either cultured alone (control) or treated with EPC‐derived exosomes or treated with EPC transfected with lncRNA‐MALAT1‐targeting siRNA‐derived exosomes (Exo‐siMALAT1) or transfected with miR‐124 mimic and treated with EPC‐derived exosomes for 24 h. n = 3, ***P < 0.001 vs control, ^##^ *P* < 0.01, ^###^ *P* < 0.001 vs Exo‐siNC

We also determined the influence of miR‐124 and lncRNA‐MALAT1 on the mRNA of *MMP9* and *CTSK*, and protein levels of MMP9, CTSK, TRAP and CAR2, which are genetic markers of osteoclastic differentiation (Figure 6A‐C). The mRNA and protein levels of MMP9, CTSK, TRAP and CAR2 follow a similar pattern to that found in ITGB1. EPC‐derived exosomes increase mRNA expression and protein levels, whereas overexpressing miR‐124 or silencing lncRNA‐MALAT1 decrease levels. These results demonstrate that exosomes induce osteoclastic differentiation through the lncRNA‐MALAT1 associated inhibition of miR‐124.

### 3.5 EPC‐derived exosomes promote the homing and osteoclastic differentiation of transplanted BMMs and further accelerate bone healing through lncRNA‐MALAT1

**Figure 7:**
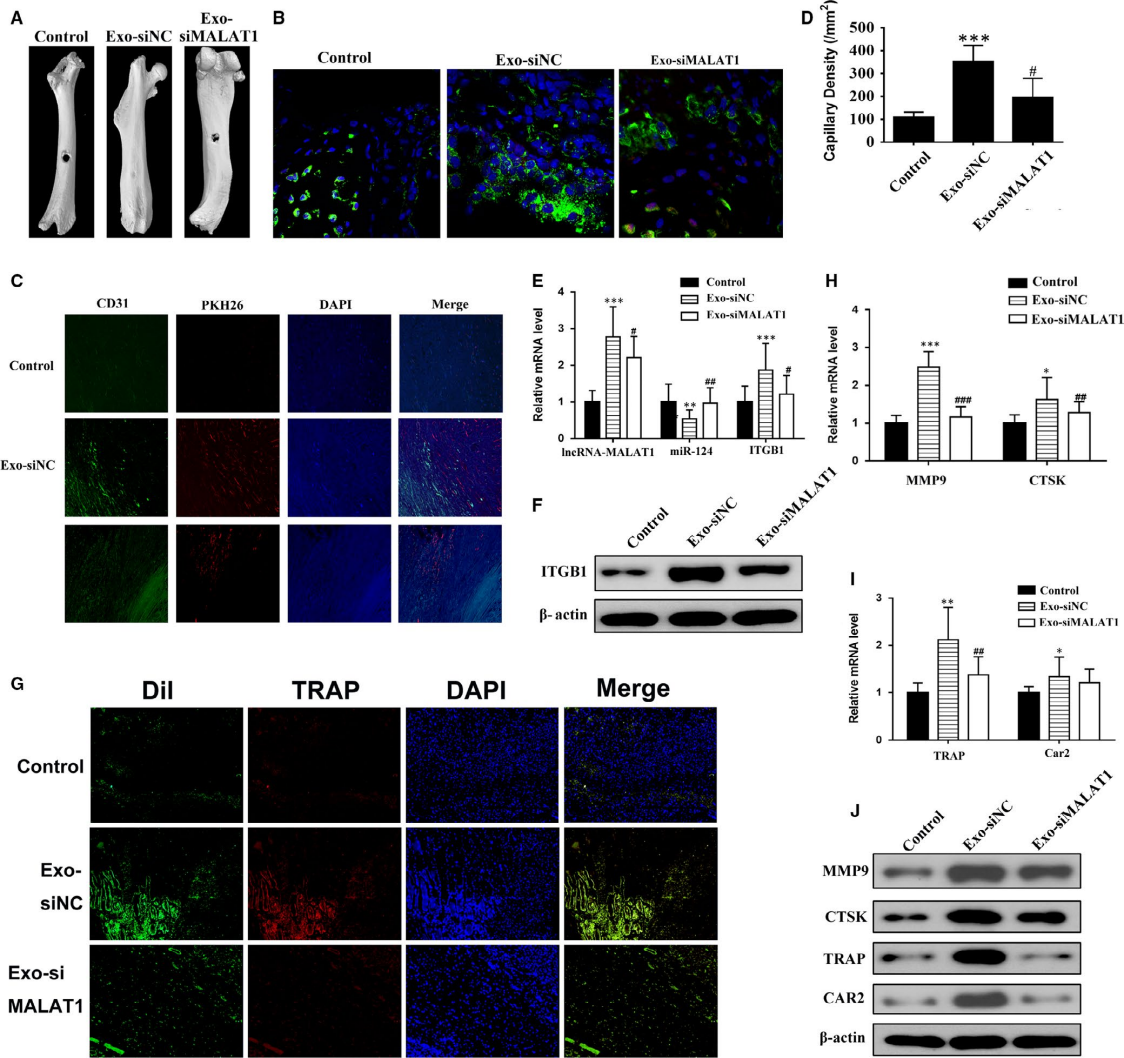
Endothelial progenitor cells (EPCs)‐derived exosomes promote the homing and osteoclastic differentiation of transplanted bone marrow‐derived macrophages (BMMs) and further accelerate bone healing through lncRNA‐MALAT1. Mice received an intravenous transplantation of Dil‐labelled BMMs, either alone (control) or treated with EPC transfected with negative control‐derived exosomes (Exo‐siNC) or with EPC transfected with lncRNA‐MALAT1‐targeting siRNA‐derived exosomes (Exo‐siMALAT1) for 24 h immediately after femur fracture. A, Representative μCT images of the fractured femur at week 4. B, Immunofluorescence staining for ITGB1 expression in Dil‐labelled BMMs around the fracture site at day 7. C, Representative fluorescence images of exosomes labelled with PKH26 and immunofluorescence images of CD31 of tissues at the fracture site at week 4. Tissues were counterstained with DAPI. D, Density of Dil‐positive capillaries at the fracture site at week 4. E, The mRNA levels of lncRNA‐MALAT1, miR‐124 and *ITGB1* (F) Protein levels of ITGB1; G, Representative fluorescence images of TRAP staining in Dil‐labelled BMMs around the fracture site at week 4; (H, I) mRNA levels of MMP9, CTSK, TRAP and CAR2; J, western blot analysis of MMP9, CTSK, TRAP and CAR2 in tissues at the fracture site at day 7. n = 3; **P < 0.01, ***P < 0.001 vs control; # *P* < 0.01, ##*P* < 0.01, ###*P* < 0.001 vs Exo‐siMALAT1

Finally, we assessed the bone healing effectiveness of EPC‐derived exosomes in a mouse femur fracture model. Mice received an intravenous transplantation of Dil‐labelled BMMs, either as a control or treated with EPCs transfected with NC‐derived exosomes (Exo‐siNC) or treated with exosomes derived from EPCs transfected with lncRNA‐MALAT1‐targeting siRNA (Exo‐siMALAT1) for 24 hours immediately after the femur fracture. After 4 weeks, the femurs were recovered from killed mice and the quality of the repair was assessed. Representative μCT images of the fractured femur at week 4 indicate that the bones had healed more effectively in mice that received the Exo‐siNC treatment than in the control or in mice treated with Exo‐siMALAT1, which suggests that EPC‐derived exosomes promote osteoclastic differentiation, whereas the silencing of lncRNA‐MALAT1 inhibits osteoclastic differentiation in vivo (Figure 7A). Similarly, immunofluorescence staining of ITGB1 expression in Dil‐labelled BMMs around the fracture site at day 7 shows that there is a greater intensity of Dil‐labelled BMMs expressing ITGB1 at the site of injury in the Exo‐siNC‐treated mice compared to the control or Exo‐siMALAT1‐treated mice (Figure 7B). Representative fluorescence images of the exosome labelled with PKH26 and CD31, which is an endothelial cell marker used to detect exosome maturation and target cell binding, are clearly present at the fracture site of Exo‐siNC‐treated mice after 4 weeks (Figure 7C). Moreover, the density of Dil‐positive capillaries at the fracture site at week 4 was significantly greater in Exo‐siNC‐treated mice (*P* < 0.001 vs control, *P* < 0.01 vs Exo‐siMALAT1‐treated mice) (Figure 7D). The mRNA levels of lncRNA‐MALAT1, miR‐124 and ITGB1 were evaluated in BMMs either grown alone or treated with Exo‐siNC or Exo‐siMALAT1. RT‐PCR results showed that the expression of lncRNA‐MALAT1 and ITGB1 was elevated in BMMs treated with Exo‐siNC compared with BMMs treated with Exo‐siMALAT1 or the control. However, levels of miR‐124 decreased (Figure 7E). ITGB1 protein levels were also increased in Exo‐siNC‐treated mice (Figure 7F). Representative fluorescence images of TRAP staining in Dil‐labelled BMMs around the fracture site at week 4 revealed that osteoclastic differentiation was less induced by Exo‐siMALAT1 than by siNC (*P* < 0.001 vs Exo‐siNC) (Figure 7G). The mRNA and protein levels of MMP9, CTSK, TRAP and CAR2 were all elevated in mice treated with Exo‐siNC compared with mice treated with Exo‐siMALAT1 or control mice (Figure 7H‐J).

The overall results indicated that EPC‐derived exosomes play a vital role in promoting the homing and osteoclastic differentiation of transplanted BMMs, and further accelerate bone healing, possibly through the inhibition of miR‐124 by LncRNA‐MALAT1.

## 4 DISCUSSION

This study aimed to explain the mechanisms of non‐coding RNA on the induction of osteoclast formation and function by EPCs. The effects of EPC‐derived exosomes on the migration and osteoclastic differentiation of primary mouse BMMs were examined in vitro. We also evaluated the effects of EPC‐derived exosomes on the homing and osteoclastic differentiation of transplanted BMMs in a mouse bone fracture model in vivo. We were able to show that EPCs secreted exosomes containing lncRNA‐MALAT1 in EPC‐BMM co‐culture medium. Moreover, the exosomes derived from EPCs showed a higher expression level of lncRNA‐MALAT1. We confirmed that lncRNA‐MALAT1 could directly bind to miR‐124 and propose that lncRNA‐MALAT1 could act as a sponge to negatively control miR‐124 activity.

Several studies have suggested that lncRNAs can base pair with miRNAs, thereby, effectively depleting them by acting as a sponge or decoy.^29^, ^30^, ^31^ For instance, a recent study found that microRNA‐487b was a direct target of lncRNA muscle anabolic regulator 1 (MAR1).^32^ LncRNA MAR1 acts as a miR‐487b sponge to regulate the Wnt5a protein, which results in the promotion of muscle differentiation and regeneration. Moreover, other studies have also found that these LncRNA are enriched in exosomes^33^, ^34^ and may even provide new diagnostic and prognostic markers in a tumour environment.^35^

In the present study, we found that EPC‐derived exosomes increase the mRNA expression of MMP9, CTSK, TRAP and CAR2, genes associated with osteoclastic differentiation, whereas overexpressing miR‐124 or silencing lncRNA‐MALAT1 decreased the expression of these genes. It has been suggested that osteoclasts stimulate angiogenesis by the secretion of MMP‐9.^36^ MMP‐9 is thought to be important in osteoclast invasion of the long bone growth plate and VEGF‐induced osteoclast migration.^37^ CTSK, used as a marker of osteogenesis in this study, is a cysteine protease that is secreted by osteoclasts to degrade matrix collagen.^38^ More importantly, CTSK activates TRAP, which is highly expressed in osteoclasts where it initiates the dephosphorylation of bone matrix phosphoproteins.^39^ The use of TRAP as a molecular marker has allowed the identification of several miRNAs involved in osteoclastogenesis processes, including miR‐124.^40^ Previous studies have indicated that miR‐124 may negatively regulate osteoclast differentiation by suppressing the expression of NFATc1, a key regulator of osteoclastogenesis.^19^, ^41^ In agreement with our results, the expression of miR‐124 was found to decrease during osteoclastic differentiation, moreover, inhibition of miR‐124 was found to promote NFATc1 expression and osteoclastogenesis.^19^

In this study, we also discovered that there was a potential miR‐124 binding site in the 3′‐UTR of the ITGB1, and an interaction between miR‐124 and ITGB1 was confirmed through a luciferase reporter assay. The mRNA levels of miR‐124 and ITGB1 were measured by quantitative RT‐PCR. When levels of miR‐124 mRNA increase, levels of ITGB1 are reduced. Integrins are involved in several functions associated with osteoclastogenesis, including substrate recognition, cytoskeletal organization and matrix‐derived signalling.^42^ The failed activation of β1 integrins is associated with osteoclast dysfunction.^43^

Bone defects are normally treated with autologous bone grafting. However, this approach has limitations due to the problems associated with retrieving bone tissue from a second site. Bioactive scaffolds are being adopted as an alternative solution to allow the attachment and differentiation of transplanted cells.^44^ In this study, we have exploited the exosomal location of lncRNA‐MALAT1 in EPCs to promote the osteoclastic differentiation of BMMs. Mice treated with EPC‐derived exosomes and BMMs exhibited increased neovascularization at the fracture site and enhanced fracture healing compared with those treated with BMMs alone. Therefore, EPC‐derived exosomes in combination with BMMs could have potential as an osteogenic factor in a bioactive scaffold.

To conclude, we suggested that EPC‐derived exosomes could promote osteoclastogenesis through the LncRNA‐MALAT1/miR124 pathway. Our present findings demonstrate that exosomes derived from EPCs have a higher expression level of LncRNA‐MALAT1 than EPCs. LncRNA‐MALAT1 can directly bind to miR‐124 to negatively control miR‐124 activity. This, in turn, may regulate levels of integrins, such as ITGB1, which play an important role in osteoclastogenesis through substrate recognition, cytoskeletal organization and matrix‐derived signalling. We also demonstrated that EPC‐derived exosomes expressing LncRNA‐MALAT1 could be delivered successfully to a bone fracture site in an animal model to increase neovascularization. Overall, our results suggest that EPC‐derived exosomes can promote bone repair in vivo by enhancing recruitment and differentiation of osteoclast precursors through controlling levels of miR‐124 via LncRNA‐MALAT1.

## CONFLICT OF INTEREST

The authors declare that they have no conflicts of interests.
